# Constructing an Associative Memory System Using Spiking Neural Network

**DOI:** 10.3389/fnins.2019.00650

**Published:** 2019-07-03

**Authors:** Hu He, Yingjie Shang, Xu Yang, Yingze Di, Jiajun Lin, Yimeng Zhu, Wenhao Zheng, Jinfeng Zhao, Mengyao Ji, Liya Dong, Ning Deng, Yunlin Lei, Zenghao Chai

**Affiliations:** ^1^Institute of Microelectronics, Tsinghua University, Beijing, China; ^2^School of Computer Science and Technology, Beijing Institute of Technology, Beijing, China

**Keywords:** spiking neural network, artificial intelligence, associative memory system, Hebb's rule, STDP

## Abstract

Development of computer science has led to the blooming of artificial intelligence (AI), and neural networks are the core of AI research. Although mainstream neural networks have done well in the fields of image processing and speech recognition, they do not perform well in models aimed at understanding contextual information. In our opinion, the reason for this is that the essence of building a neural network through parameter training is to fit the data to the statistical law through parameter training. Since the neural network built using this approach does not possess memory ability, it cannot reflect the relationship between data with respect to the causality. Biological memory is fundamentally different from the current mainstream digital memory in terms of the storage method. The information stored in digital memory is converted to binary code and written in separate storage units. This physical isolation destroys the correlation of information. Therefore, the information stored in digital memory does not have the recall or association functions of biological memory which can present causality. In this paper, we present the results of our preliminary effort at constructing an associative memory system based on a spiking neural network. We broke the neural network building process into two phases: the Structure Formation Phase and the Parameter Training Phase. The Structure Formation Phase applies a learning method based on Hebb's rule to provoke neurons in the memory layer growing new synapses to connect to neighbor neurons as a response to the specific input spiking sequences fed to the neural network. The aim of this phase is to train the neural network to memorize the specific input spiking sequences. During the Parameter Training Phase, STDP and reinforcement learning are employed to optimize the weight of synapses and thus to find a way to let the neural network recall the memorized specific input spiking sequences. The results show that our memory neural network could memorize different targets and could recall the images it had memorized.

## 1. Introduction

Development of computer science has led to the blooming of artificial intelligence (AI). Research on AI has become extremely popular these days due to the ever-growing demands from application domains such as pattern recognition, image segmentation, intelligent video analytics, autonomous robotics, and sensorless control (Rowley et al., 1996; Lecun et al., 1998; Zaknich, 1998; Egmont-Petersen et al., 2002). Neural networks are the core of AI research. Deep-learning neural networks (DNNs), the second generation of artificial neural networks (ANNs), have become the research hotspot of neural networks (Schmidhuber, 2014) and have won numerous contests against people, including the most famous one: recently, Google's AlphaGo DNN defeated Lee Sedol, a famous professional I-go player.

To date, many studies have been conducted on DNN, focusing on development of the learning and training methods (Jennings and Wooldridge, 2012; Yoshua et al., 2013; Lecun et al., 2015) of DNN. Researchers studying DNN typically use a fixed neural network structure and train their DNN using a large amount of data to optimize the weight of the connections/synapses.

Although the mainstream neural networks have done well in the fields of image processing and speech recognition, they do not perform well in models aimed at understanding contextual information. In our opinion, the reason for this is that the essence of building a neural network through parameter training is to fit the data to the statistical law through parameter training. Since the neural network built using this approach does not possess memory ability, it cannot reflect the relationship between data with respect to the causality. Recurrent neural networks (RNNs) use a special network structure to address this issue, but the complexity of its structure also leads to many limitations.

Spiking neural networks (SNNs) are the third generation of ANNs. Compared with DNNs, SNNs are more similar to the biological neural network; SNNs use spiking neurons, which emit spiking signals when activated. The generated spiking trains (sequences of spiking signals) are used to communicate between neurons. Spiking train expresses time dimension information naturally; therefore, SNNs offer an advantage when dealing with information having string contextual relevance. However, due to the lack of effective training algorithms, SNNs have not yet been applied to many domains. Many studies on SNNs have been published, but most of these involve using SNNs to perform simple classification or image recognition.

Neural networks in organisms can perform many complex functions, including memory. Since SNNs are more similar to the biological neural network, we endeavored to use it to construct a bionic memory neural network. Biological memory is fundamentally different from the current mainstream digital memory in terms of the storage method. The information stored in digital memory is converted to binary code and written in separate storage units. This physical isolation destroys the correlation of information. Therefore, the information stored in digital memory does not have the recall or association functions of biological memory which can present causality.

The great capability and potential of biological neural network fascinates us. So in this paper, we present our preliminary effort at constructing an associative memory neural network based on SNN. We present our method which could guide the grow process of the memory neural network. We present our method to optimize the weight of synapses of the neural network. And through our experimental results, we show that the memory neural network built using our method could possess memory and recall ability after only undergoing a small scale of training.

In our method, we broke the neural network building process into two phases: the Structure Formation Phase and the Parameter Training Phase. The Structure Formation Phase applies a learning method based on Hebb's rule to provoke neurons in the memory layer to new synapses to connect to neighbor neurons as a response to the specific input spiking sequences fed to the neural network. The aim of this phase is to train the neural network to memorize the specific input spiking sequences. During the Parameter Training Phase, STDP and reinforcement learning are employed to optimize the weight of synapses and thus find a way let the neural network recall the memorized specific input spiking sequences.

The remaining text is organized as follows: section 2 discusses related work, section 3 mentions our motivation, section 4 provides the study background, and section 5 discusses our method to implement the memory neural network; the experimental results are reported and discussed in section 6. The conclusion is provided in section 7.

## 2. Related Works

Neural network construction has a long history, and many algorithms have been proposed (Śmieja, 1993; Fiesler, 1994; Quinlan, 1998; Perez-Uribe, 1999).

As the second generation of ANNs, DNNs have many advantages. However, they rely heavily on data for training. With the construction of DNN becoming increasingly complex and powerful, the training process requires an increasing number of computations, which has become a great challenge. Each session of training becomes increasingly time and resource consuming, which may become a bottleneck for DNNs in the near future. Now, an increasing number of researchers are turning their attention to SNNs.

In 2002, Bohte et al. (2000) derived the first supervised training algorithm for SNNs, called SpikeProp, which is an adaptation of the gradient-descent-based error-back-propagation method. SpikeProp overcame the problems inherent to SNNs using a gradient-descent approach by allowing each neuron to fire only once (Wade et al., 2010). In 2010, Wade et al. presented a synaptic weight association training (SWAT) algorithm for spiking neural networks (SNNs), which merges the Bienenstock-Cooper-Munro (BCM) learning rule with spike timing dependent plasticity (STDP) (Wade et al., 2010).

In 2013, Kasabov et al. (2013) introduced a new model called deSNN, which utilizes rank-order learning and Spike Driven Synaptic Plasticity (SDSP) spike-time learning in unsupervised, supervised, or semi-supervised modes. In 2017, they presented a methodology for dynamic learning, visualization, and classification of functional magnetic resonance imaging (fMRI) as spatiotemporal brain data (Kasabov et al., 2016). The method they presented is based on an evolving spatiotemporal data machine of evolving spiking neural networks (SNNs) exemplified by the NeuCube architecture (Kasabov, 2014), which adopted both unsupervised learning and supervised learning in different phases.

In 2019, He et al. (2019) proposed a bionic way to implement artificial neural networks through construction rather than training and learning. The hierarchy of the neural network is designed according to analysis of the required functionality, and then module design is carried out to form each hierarchy. The results show that the bionic artificial neural network built through their method could work as a bionic compound eye, which can achieve the detection of an object and its movement, and the results are better on some properties, compared with the Drosophila's biological compound eyes.

Some studies have already attempted to design neural networks that behave similar to a memory system. Lecun et al. (2015) proposed RNNs for time domain sequence data; RNNs use a special network structure to address the aforementioned issue, but the complexity of their structure also leads to many limitations.

Hochreiter and Schmidhuber (1997) presented the long short-term memory neural network, which is a variant of RNNs. This neural network inherits the excellent memory ability of RNNs with regard to the time series and overcomes the limitation of RNN, that is, difficulty in learning and preserving long-term information. Moreover, it has displayed remarkable performance in the fields of natural language processing and speech recognition. However, the efficiency and scalability of long short-term memory is poor.

Hopfield (1988) has established the Hopfield network, which is a recursive network computing model for simulating a biological neural system. The Hopfield network can simulate the memory and learning behavior of the brain. The successful application of this network to solve the traveling salesman problem shows the potential computing ability of the neural computing model for the NP class problem. However, the network capacity of the Hopfield network model is determined by neuron amounts and connections within a given network, thus the number of patterns that the network can remember is limited. Also, since patterns that the network uses for training (called retrieval states) become attractors of the system, repeated updates would eventually lead to convergence to one of the retrieval states. Thus, sometimes the network will converge to spurious patterns (different from the training patterns). And when the input patterns are similar, the network cannot always recall the correct memorized pattern, which means the fault-tolerance is affected by the relationship between input patterns.

## 3. Motivation

**Figure 1:**
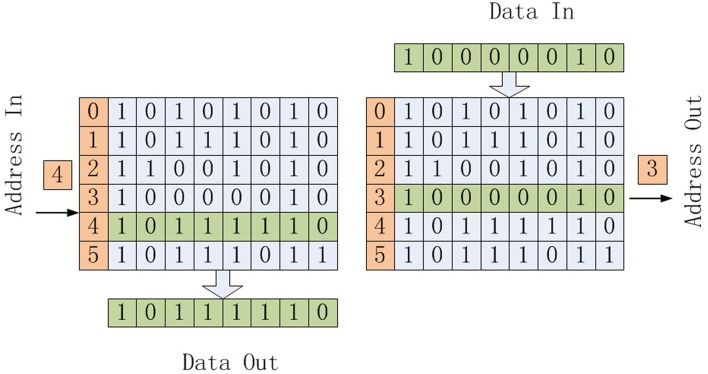
Traditional Memory and CAM.

In traditional memory, as shown in the left part of Figure 1, when we input an address, the memory outputs data stored in that address. In content addressable memory (CAM), as shown in the right part of Figure 1, when we input data, the address of that data is outputted.

**Figure 2:**
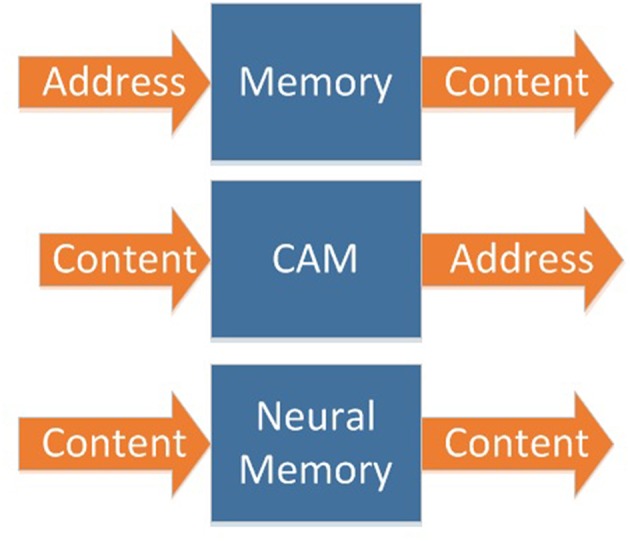
Three different mechanism of memory.

**Figure 3:**
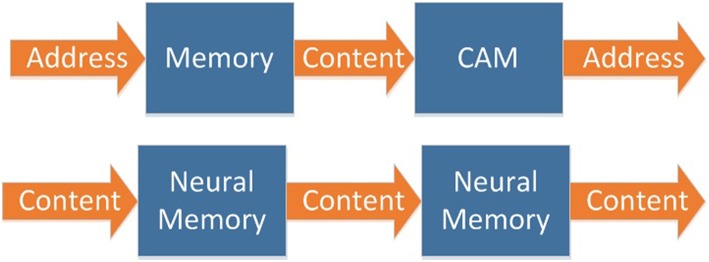
Memory's cascading mechanism.

In biological memory systems, both input and output are contents (Figure 2). Traditional memory and CAM can be cascaded to expand, as shown in Figure 3. However, due to the designing and addressing method of CAM, it is difficult to implement very large scale CAM. So, it is not able to implement cascaded CAM with large capacity in this way.

Biological memory systems are built on a neural network, which is composed of neurons. This kind of memory has a simple structure, large capacity, and can be easily expanded to a very large scale (Figure 3).

Therefore, the goal of this study was to build a bionic memory neural network.

## 4. Background

### 4.1. Neuron Model

The leaky integrate and fire neuron model was used in this study (Indiveri, 2003). It is one of the most widely used models due to its computing efficiency. This model's behavior can be described as Equation 1.

$$V(t)=\{\begin{array}{l} \beta \;\cdot \;V(t-1)+V_{in}(t)\;when\;V<V_{th}\\ V_{reset}\;and\;set\;a\;spike\;when\;V\geq V_{th} \end{array}$$

where *V*(*t*) is the state variable and β is the leaky parameter; *V*_*th*_ is the threshold state and *V*_*reset*_ is the reset state. Once *V*(*t*) exceeds the threshold *V*_*th*_, the neuron fires a spike and *V*(*t*) is reset to *V*_*reset*_.

### 4.2. Spiking Neural Networks

SNNs are inspired by the manner in which brain neurons function: through synaptic transmission of spiking trains. Spiking encoding integrates multiple aspects of information, such as time, space, frequency, and phase. It is an effective tool for complex space-time information processing. In addition, because SNNs contain time dimension information, its information processing ability is stronger than that of the previous two generations of neural networks, especially in the processing of information with strong contextual relevance.

There are many kinds of SNNs. In SNNs, all the information is encoded in spiking signals. Spiking trains, consisting of sequences of spiking signals, are transmitted in the neural network to implement communication between neurons.

### 4.3. Spike-Timing-Dependent Plasticity

Spike-timing-dependent plasticity (STDP) is one of the most important unsupervised learning rules in the SNNs. As a biological process, it describes the regulatory mechanism of synapses between neurons in the brain. In our method, STDP is used to guide the adjustment of the weight of synapses during the training of SNNs.

Let us suppose that there is a synapse from neuron *N*_*pre*_ to neuron *N*_*suc*_ in an SNN, and the firing time of *N*_*pre*_ is *t*_1_ while that of *N*_*suc*_ is *t*_2_. According to STDP, if *t*_1_<*t*_2_, then the weight of the synapse from *N*_*pre*_ to *N*_*suc*_ should increase; if *t*_1_>*t*_2_, then the weight of the synapse from *N*_*pre*_ to *N*_*suc*_ should decrease; if *t*_1_ = *t*_2_, then nothing should happen. The value of the increase/decrease in weights depends on the difference between *t*_1_ and *t*_2_.

### 4.4. Hebb's Learning Rule

The structure of a biological neural network is neither regular nor completely disordered, which is the result of the reflection to the input spiking sequences it receives. Or, we can say that it is the input spiking signals that define the structure of a biological neural network through learning and training. For example, in biological auditory systems, the structure of neural networks is related to their sensitivity to different frequencies of sound. However, the relationship between network structure and external stimulation is difficult to describe using a mathematical formula.

In our algorithm, we have applied a learning method based on Hebb's rule to form the structure of the memory neural network as a response or reflection of the input spiking sequences. Hebb's learning rule (Hebb, 1988) is a neuropsychological theory put forward by Donald Hebb in 1949. According to Hebb's learning rule (Hebb, 1988), when an axon of cell A is sufficiently close to excite a cell B, and repeatedly or persistently takes part in firing it, some growth-related process or metabolic changes take place in one or both cells such that A's efficiency, as one of the cells firing B, is increased.

## 5. Method to Construct Bionic Memory Neural Network

Our method for constructing the bionic memory neural network consists of four major phases:

1. Initialization phase: Initialize the input spiking sequences and initialize the neural network;
2. Structure Formation phase: Applies a learning method based on Hebb's rule to provoke neurons in the memory layer growing new synapses to connect to neighbor neurons as a response to the specific input spiking sequences fed to the input layer of the neural network, until the connection between the memory layer and the output layer is completed;
3. Parameter Training phase: STDP and reinforcement learning are employed to optimize and adjust the weight of synapses in the neural network;
4. Pruning phase: Comply with biological rules to delete unnecessary connections, thus enhancing the energy efficiency.

The detail process of our method is described in Algorithm 1.

**Algorithm 1:** Experiment Process

**Input:** Input Image Set, *S*; Original Memory Neural Network, *NN*;
**Output:** Trained Memory Neural Network, *NN*;
1: Initialize the Input Spiking Sequences by employing the data preprocessing process to convert *S* into spiking sequences set *SS*;
2: Initialize the memory neural network;
3: Set the turn mark of the Structure Formation phase, *TM*_*SF*_ = 0;
4: **while** (*TM*_*SF*_ < 2) **do**
5: Set the training set of the Structure Formation phase, *S*_1_ = *SS*;
6: **while** (*S*_1_≠ϕ) **do**
7: Pick one input spiking sequence *R* from *S*_1_, and delete it from *S*_1_;
8: Feed *R* to the memory neural network, and perform Structure Formation phase;
9: **end while**
10: *TM*_*SF*_ = *TM*_*SF*_+1;
11: **end while**
12: Set the training set of the Parameter Training phase, *S*_2_ = *SS*;
13: **while** (*S*_2_≠ϕ) **do**
14: Pick one input spiking sequence *R* from *S*_2_;
15: Feed *R* to the memory neural network;
16: **if** Result of the output layer is correct **then**
17: Delete *R* from *S*_2_;
18: **else**
19: Perform the Parameter Training phase for *R*;
20: **end if**
21: **end while**

In this work, the MNIST dataset (Lecun and Cortes, 2010) was selected to test our proposed method. The MNIST is a widely used dataset for optical character recognition, with 60,000 handwritten digits in the training set and 10,000 in the testing set. The size of handwritten digital images in this dataset is 28 × 28.

As stated in Algorithm 1, during the parameter training phase, we would test the memory neural network if it could recall the image it has already memorized. We would present one image from MNIST (already been processed and transferred into spiking sequence) to the input layer for a certain time duration. The input spiking sequence would be transferred to the memory layer. Neurons in the output layer would receive responses from the memory layer and fire if necessary, thus we could record the firing sequence from the output layer. Since one image would only be fed to the input layer for a limited time duration, after a while, there would be no more firing in the output layer, which indicates the end of the firing sequence. Then we will decide the meaning of this firing sequence by the majority votes method.

### 5.1. Initialization Phase

#### 5.1.1. Initialize the Input Spiking Sequences

Since the input to our memory neural network should be spiking sequences, the MNIST images should first be transferred into the spiking sequence. When the input spiking sequences are initialized, a data preprocessing process is designed to convert the MNIST images into spiking sequences.

**Figure 4:**
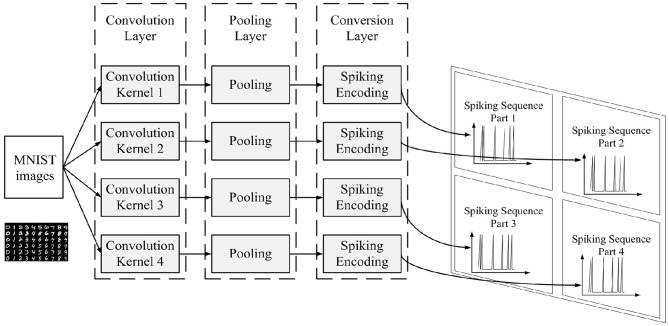
Data preprocessing process for initializing MNIST images into input spiking sequences.

The data preprocessing process is shown in Figure 4.

**Figure 5:**
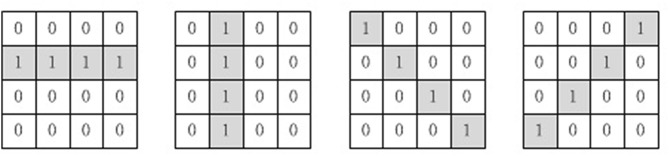
Four convolution kernels used in our method.

The convolution layer and the pooling layer are added to abstract the features of the MNIST images, thus reducing the amount of information our memory neural network needs to memorize. Four 4 × 4 convolution kernels are used in the convolution layer, which are shown in Figure 5. MNIST images would be first processed by the four convolution kernels separately, then the result of the four convolution kernels would be processed by the pooling layer. The pooling layer employs 2 × 2 max_pooling operation.

The conversion layer is used to convert the images outputted by the pooling layer into spiking sequences according to the spiking encoding method. There are many kinds of encoding methods in literature. The principle of priority transmission of important information in the ROC (Rank Order Coding) coding method (Thorpe and Gautrais, 1998) is used to help design the encoding method in this paper. The spiking encoding method used in this paper converts the pixel value of the image into the delay time of the spiking signal, and the higher the pixel value is, the shorter the delay time is.

Suppose the set of pixels in an image is *D*, then for each pixel *d* ∈ *D*, min_max normalization would first be employed to avoid the singular sample data affecting the convergence of the network:

(1) $$\begin{array}{r} R\left(d\right)=\frac{d-d_{min}}{d_{max}-d_{min}} \end{array}$$

where *d*_*max*_ and *d*_*min*_ are the maximum and minimum value in *D*, respectively.

Four different spiking encoding methods have been designed in this paper:

Method 1: Linear encoding method, where *S*(*d*) = *T*_*max*_−*R*(*d*) × (*T*_*max*_−*T*_*min*_);
Method 2: Exponential encoding method, where $S\left(d\right)=\left(0.5^{R\left(d\right)-1}-1\right)\times \left(T_{max}-T_{min}\right)+T_{min}$;
Method 3: Inverse encoding method, where $S\left(d\right)=\left(\frac{2}{R\left(d\right)+1}-1\right)\times \left(T_{max}-T_{min}\right)+T_{min}$;
Method 4: Power encoding method, where $S\left(d\right)={\left(R\left(d\right)-1\right)}^{2}\times \left(T_{max}-T_{min}\right)+T_{min}$.

where *T*_*max*_ and *T*_*min*_ are the stop time and start time of the spiking sequence for that image, while *S*(*d*) is the converted spiking time for pixel *d*.

**Figure 6:**
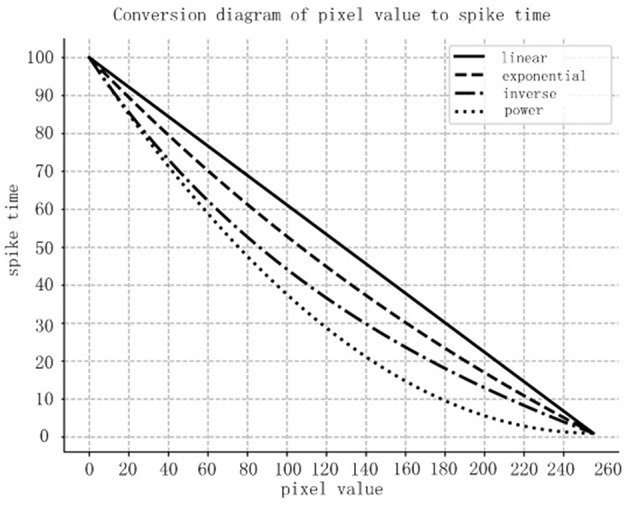
Comparison of the four encoding methods.

The relationship between the pixel value and the spiking time for those four methods is compared in Figure 6. In the graph, the horizontal coordinates represent the pixel values, while the vertical coordinates are the encoded spiking times. According to the comparison, we can conclude that the power encoding method could emit more important information in an earlier time, thus we chose the power encoding method as the spiking encoding method for this paper.

The pixel value range of the MNIST images is [0,255]. After being processed by the conversion layer, an image from the MNIST set would be converted into an input spiking sequence with spiking signals in a time range of [0, 100 ms].

#### 5.1.2. Initialize the Neural Network

**Figure 7:**
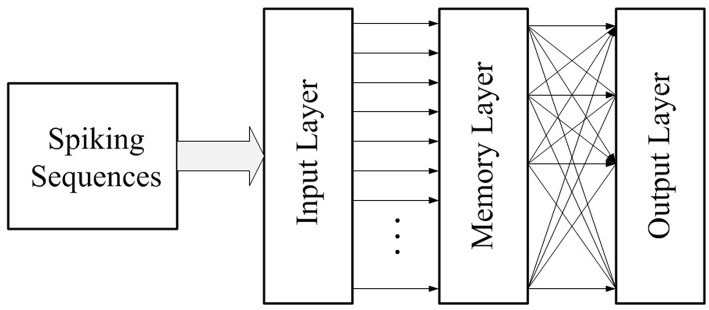
Structure of our memory neural network.

Our memory neural network consists of three layers: the input layer, the memory layer and the output layer, as shown in Figure 7. The input layer is in charge of receiving input spiking sequences and feeding the input spiking sequences into the memory layer. The memory layer would grow new connections as a response of input spiking sequences to remember them, then through proper training recall them and output the correct result through the output layer. The output layer exists because we not only want our neural network to possess memory ability, but also to be able to output recall result. The number of neurons in the output layer is set as the same as the number of targets that the memory neural network needs to be memorized.

The task of this initialization phase is to initialize all three layers and initialize the connections between the input layer and the memory layer. The number of neurons in the input layer is determined by the size of the target to be memorized. As shown in **Figure 9**, neurons in the input layer are connected to neurons in the memory layer with a one-to-one style. So, the number of neurons in the memory layer is same as the input layer. The weight of synapses in this work is set in the range [0, 100]. In order to provoke enough responses in the memory layer to allow the learning method based on Hebb's rule to work, the initialized weight of connection from the input layer to the memory layer should be strong enough, and is set as 50 in this work.

Since the original MNIST image is 28 × 28, after the operation of the four convolution kernels in the convolution layer, the result is 4 parts each with sizes of 25 × 25, and after the pooling layer, the result is 4 parts each with sizes of 12 × 12. Since the result after the pooling layer is 4 parts each with sizes of 12 × 12, there are 576 spiking signals in the spiking sequence in total after the process of the conversion layer. Thus, in this work, we set 576 neurons in the input layer of our memory neural network. Each spiking signal in the spiking sequence would feed into one of the input neurons. And since the connection style between input layer and memory layer is one-to-one, there are also 576 neurons in the memory layer in this work.

**Figure 8:**
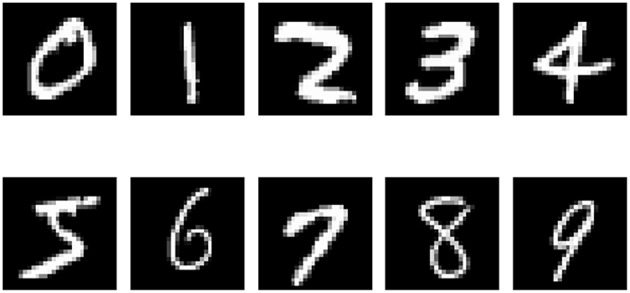
Input Image Set *S*.

Ten images, each of different number (that is one image of each from 0 to 9), are chosen from MNIST to form the Input Image Set *S* of this work, as shown in Figure 8. Thus the number of neurons in the output layer is 10, corresponding to the 10 images needed to be memorized.

**Figure 9:**
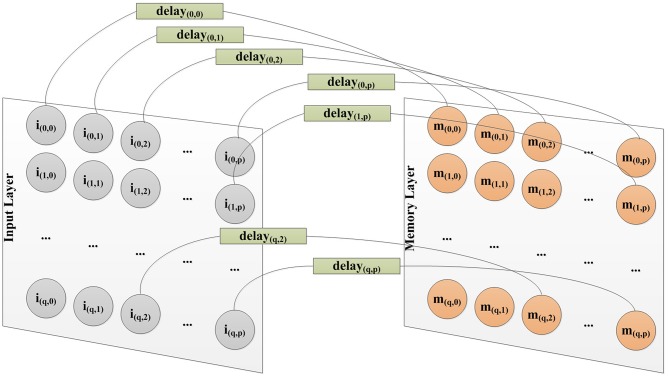
Different delay for each connection from input layer to memory layer to capture spatial information.

Each neuron in the input layer and memory layer will be assigned a coordinate, as shown in Figure 9. The coordinates of neurons in the memory layer would be used to calculate the distance between them in later course of our algorithm.

As stated before in the paper, we use MNIST images as the input. There are two kinds of information in a Mnist image. The value of the pixel, and the location of that pixel. We use the power encoding method to convert the value of the pixel into the spiking time of that pixel. And in order to capture the spatial information of the pixels, we have implemented a spatial-to-temporal mechanism to decide the delay of a connection from neurons in the input layer to neurons in the memory layer, as shown in Figure 9. The delay of a connection from neuron *i*_(*x, y*)_ in a *p*×*q* input layer to neuron *m*_(*x, y*)_ in a *p*×*q* memory layer is calculated as:

(2) $$\begin{array}{r} delay_{im\left(x,y\right)}=x*p+y+1 \end{array}$$

here (*x, y*) is the coordinate of that neuron.

This acts as a way to encode spatial information into temporal information, which then could be captured by SNNs.

### 5.2. Structure Formation Phase

During the structure formation phase, input spiking sequences would be fed to the input layer of the memory neural network, which would then be fed to the memory layer through connections between the input layer and the memory layer. The behavior of all the neurons in the memory layer would be recorded. Additionally, a learning method is conducted to direct the growing of new connections in the memory layer.

According to Hebb's learning rule (Hebb, 1988), when an axon of cell A is sufficiently near to excite a cell B, and repeatedly or persistently takes part in firing it, some growth-related process or metabolic changes take place in one or both cells such that A's efficiency, as one of the cells firing B, is increased.

A learning method based on Hebb's learning rule is designed to direct the growing of new connections (synapses) in the structure formation phase. According to our learning algorithm, if the firing times of two neurons are very close, and there is no connection between them, a connection is established between them. In order to prevent the explosive growth of network connections, our approach considers the coordinate of neurons and does not establish connections when the Euclidean distance between neurons exceeds a pre-defined threshold.

The detail description of this algorithm is provided below:

Step 1: Start the simulation, record firing behaviors of neurons in the memory layer;
Step 2: Examine whether there exists a pair of neurons *N*_1_ and *N*_2_ in the memory layer such that both have fired during the simulation, and the distance between neurons *N*_1_ and *N*_2_ satisfies that *Dis*(*N*_1_ *to N*_2_) < *Dis*_*threshold*_ (where *Dis*_*threshold*_ is a pre-defined distance threshold for our algorithm). If any, proceed to Step 3; otherwise, proceed to Step 4;
Step 3: Suppose the firing time of *N*_1_ is *t*_1_, and that of *N*_2_ is *t*_2_. If 0 < *abs*(*t*_1_−*t*_2_) < *Threshold* and (*t*_1_ < *t*_2_), establish a connection from *N*_1_ to *N*_2_ with weight of 10, and proceed to Step 4; if 0 < *abs*(*t*_1_−*t*_2_) < *Threshold* and (*t*_1_ > *t*_2_), establish a connection from *N*_2_ to *N*_1_ with weight of 10, proceed to Step 4; if *abs*(*t*_1_ − *t*_2_) ≥ *Threshold*, proceed to Step 4;
Step 4: If the stop criterion is satisfied, end the simulation; otherwise, go to Step 2.

Since the connections in the memory layer are grown under guidance of the learning method based on Hebb's learning rule, the distance threshold *Dis*_*threshold*_ is used to control the number of connections generated in the memory layer. If the threshold is smaller, then there would be less connections. If the threshold is larger, there would be more connections. The *Dis*_*threshold*_ in this work is set as 2.

This process continues until the stop criterion is satisfied. Then, neurons in the memory layer are connected to the neurons in the output layer according to their firing behavior. As we have discussed in section 5.1, a spatial-to-temporal mechanism has been introduced to decide the delay of a connection from input layer to memory layer, since the neuron model we used is a LIF model. In order to avoid the unnecessary reduction of firing activity of neurons in the output layer, due to the leaking characteristics of the LIF model, we have also implemented a temporal-to-spatial mechanism to calculate the delay of connection from neurons in the memory layer to neurons in the output layer. The delay of a connection formed between neuron *m*_(*x, y*)_ in the memory layer and neuron *o*_*z*_ in the output layer is calculated as:

(3) $$\begin{array}{r} delay_{mo\left(x,y\right)}=\left[N_{m}-delay_{im\left(x,y\right)}\right]+1 \end{array}$$

where *N*_*m*_ is the total number of neurons in the memory layer, while (*x, y*) is the coordinate of neurons in the memory layer as shown in Figure 9.

In our opinion, if a neuron in the memory layer fired when we fed the input spiking sequence related to a specific target, then it has causality with the memory behavior of that specific target. Since neurons in the output layer correspond to the targets needed to be memorized, we connect neurons in the memory layer which fired when we fed the input spiking sequence related to a specific target, to the neuron in the output layer which represents that specific target. The initialized weight of a connection established this way is *weight*/*n*, where *weight* is a pre-defined constant, and *n* is the number of neurons in the memory layer which are connected to that neuron in the output layer. This is an approximate process. The weight of connections from neurons in the memory layer to neruons in the memory layer or connections from neurons in the memory layer to neurons in the output layer would be optimized during the parameter training phase.

### 5.3. Parameter Training Phase

Through structure formation phase, we have made the neural network to memorize specific targets represented by input spiking sequences. However, as a memory, we still need to have a recall mechanism. When fed the specific input spiking sequence again, which the neural network has already memorized, the memory neural network needs to recall it and output a correct result, represented by the correct behavior of the output layer. During the parameter training phase, we will rely on STDP and reinforcement learning to optimize the weight of connections (synapses) in the neural network to implement the recall mechanism. The weight of connections between the input layer and memory layer would not be optimized during this phase. In the parameter training phase, the STDP option of NEST (the evaluation platform we used for this work) is always on.

The algorithm for parameter training phase is described below:

Step 1: Pick one input from the input spiking sequences training set;
Step 2: Feed the picked input to the input layer and examine the result sequence of the output layer;
Step 3: If the result sequence of the output layer is correct, go to Step 1; Otherwise go to Step 4;
Step 4: Identify the set of incorrectly firing neurons in the output layer as *S*_*O*_ and identify the set of firing neurons in the memory layer as *S*_*M*_;
Step 5: If neuron *i* is in *S*_*M*_, and neuron *j* is in *S*_*O*_, and there is a connection from neuron *i* to neuron *j*, suppose the weight of this connection is *W*_*i, j*_, then *W*_*i, j*_ = *W*_i, j_*Shrink_*Coeff*, and go to Step 2;

During the parameter training phase, when a specific input spiking sequence is fed to the input layer to train the memory neural network, the firing behavior of the neurons in the output layer would be recorded. The label corresponding to the most frequently fired neuron in the output layer is identified as the output result for this specific input spiking sequence. If the result is correct, then we suppose the memory neural network could correctly recall. If not, optimization needs to be done to establish the right recall mechanism.

As we said before, causality is the basis on which we built our method. If a specific input spiking sequence is fed to the input layer of the memory neural network, but the most frequently fired neuron in the output layer is not the correct one, it means that some of the fired neurons in the memory layer have contributed to the result under incorrect causality and thus need to be corrected while the contribution needs to be weakened.

The algorithm would seek out those connections, and STDP and reinforcement-based methods are used to optimize the weight of those connections, as shown in the algorithm description.

### 5.4. Pruning Phase

One of the most important advantages of the biological neural network is its energy efficiency. In our method, we introduced the pruning phase to delete redundant and unnecessary connections from the trained neural network. The method examines the weight of all connections. If the weight of a connection is smaller than a pre-defined threshold (set as 3 in this work), that connection is deleted. Further, if a neuron has no output connection, all the input connections of that neuron are also deleted. The pruning phase helps enhance the energy efficiency of the neural network.

## 6. Experiment Results

### 6.1. Evaluation Framework

We built our simulation platform based on the neural simulation tool NEST (Plesser et al., 2015), which is a simulation platform specially designed for SNN research. Biological spiking neural networks are characterized by the parallel operation of thousands of spiking neurons and the exchange of information between them by spiking trains sent via synapses. This mode of functioning fits the characteristics of the message passing interface parallel mechanism in particular. NEST supports message passing interface parallelization. Further, NEST provides users a method of asynchronous multi-process concurrent execution, which makes the program execute the model asynchronously and efficiently, and automatically synchronizes the process during the simulation without user interaction. Parallel computing reduces the time required and increases the scale of operations.

We conducted two sets of experiments. In the first set of experiments, in order to show the difference between the structures of the memory layer when used to memorize different targets, we used 10 identical SNNs to train 10 different images each, numbered from “0” to “9.” In the second set of experiments, we used 1 SNN to train on all those 10 images to test the recall (with those 10 images it already memorized) and association (using an image it has not seen before) ability.

### 6.2. Results and Discussion

#### 6.2.1. Growing Process of the Memory Layer

**Figure 10:**
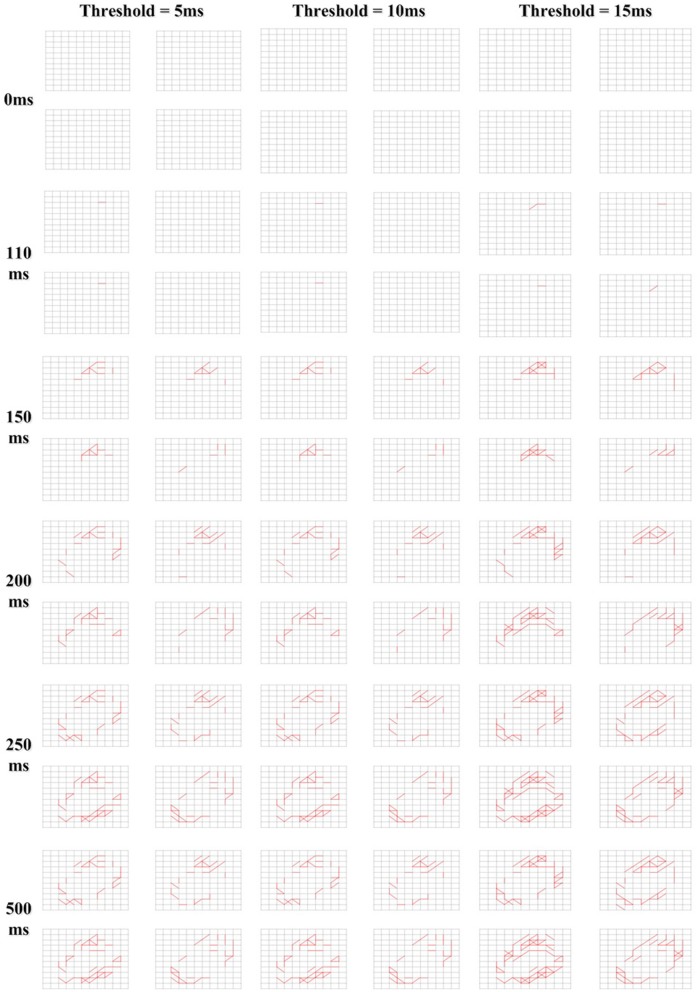
Different growing behavior due to different learning *Threshold*.

After the Initialization phase, there was no connection in the memory layer. During the Structure Formation phase, when the input spiking sequences are fed to the input layer of our memory neural network, under the control of the learning method, new connections would grow in the memory layer. An illustration of the growing process of the memory layer during Structure Transformation phase under different *Threshold* value choices is shown in Figure 10. The 4x different subpanels in each relevant panel correspond to the parts in the memory layer which are the output of each kernel. The input image is a “0” from the MNIST set. From the comparison we could conclude that, when the *Threshold* is smaller, the connection in the memory layer is more sparse, thus the memory layer could remember more due to the larger available capacity.

#### 6.2.2. Results of Memory Process

**Figure 11:**
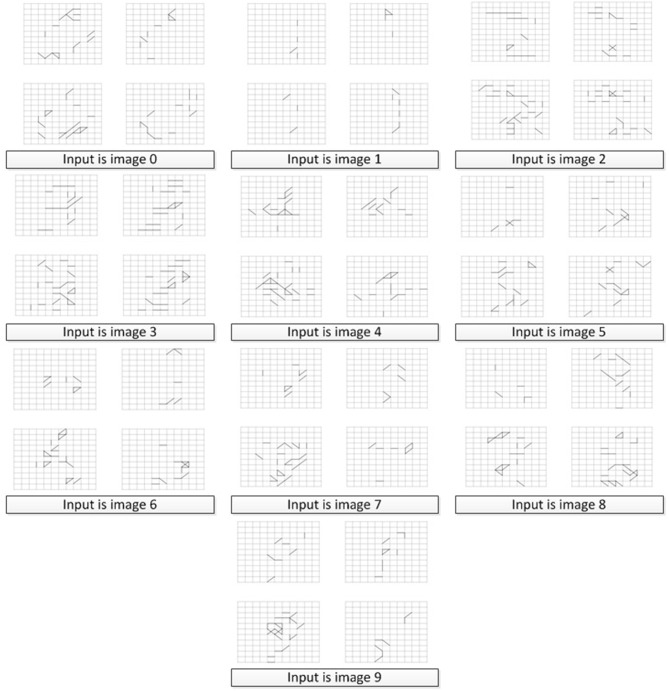
Different memory layer structure to memory different input images.

In order to verify that our memory neural network could remember different targets, we conducted the first set of experiments and built 10 memory neural networks, each fed with a different image numbered from 0 to 9 (as shown in Figure 8). The results of the memory layer after the Structure Formation phase are shown in Figure 11, and the learning *Threshold* was set to 5 ms. The 4x different subpanels in each relevant panel correspond to the parts in the memory layer which are the output of each kernel. Each memory neural network is trained with only 1 image. According to Figure 11, we could see that our memory neural network could grow different connections in the memory layer to memory different targets.

#### 6.2.3. Results of Recall Process

**Figure 12:**
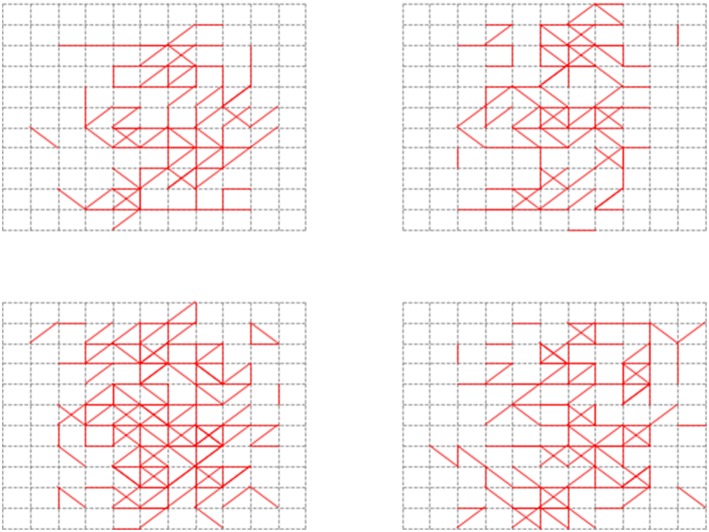
Generated memory neural network with learning *Threshold* of 5 ms.

In order to test the recall ability of our memory neural network, we conducted the second set of experiment. First, we used all the images in the Input Image Set *S* as shown in Figure 8 to perform the Structure Formation phase. Then we used the images in the Input Image Set *S* again to perform the Parameter Training phase and the Pruning phase. The memory layer of the generated memory neural network is shown in Figure 12. The 4x different subpanels in each relevant panel correspond to the parts in the memory layer which are the output of each kernel.

**Figure 13:**
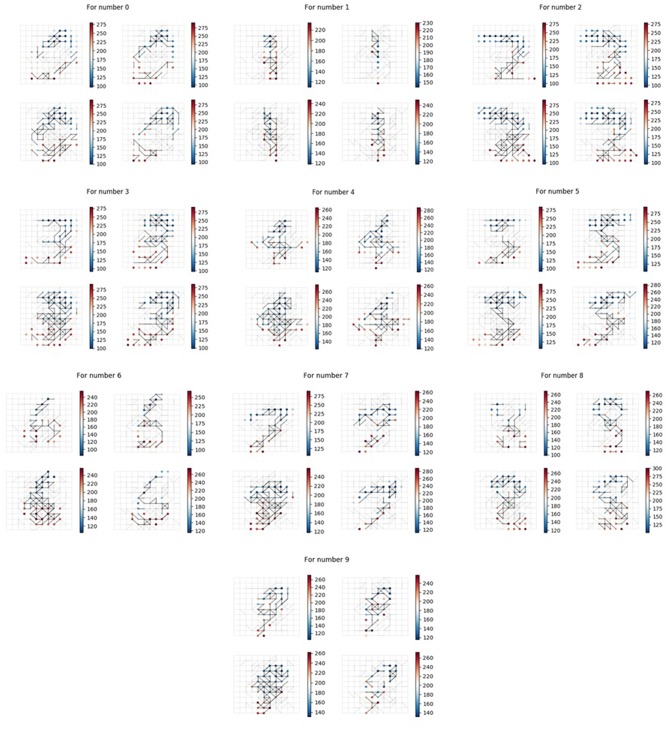
Recall response for images in the Input Image Set.

Figure 13 shows the firing behavior of the memory layer when we feed the images from the Input Image Set *S* to the generated memory neural network. The 4x different subpanels in each relevant panel correspond to the parts in the memory layer which are the output of each kernel. Different color represents different firing time, as shown in the vertical coordinate line beside each sub-figure. It could be seen that different images would provoke different parts in the memory layer to respond and generate different firing behavior. As described in section 5, when an image is fed to the memory neural network, a firing sequence of output neurons would be observed to decide the output result for that image using the majority votes method. The results are recorded in Table 1.

**Table 1:** Recall test result for memory neural network.

**Label**	**Firing sequence of output neurons**	**Output**	**Result**
0	[9 0 7 4 6 0 0 3 7 6 5 0]	0	Correct
1	[1]	1	Correct
2	[9 7 2 5 3 4 0 8 1 6 2 7 8 9 5 2 2]	2	Correct
3	[9 8 5 3 6 7 9 0 2 8 3 5 7 9 3 3]	3	Correct
4	[1 9 4 9 4 6 4 4]	4	Correct
5	[5 7 9 0 6 3 5 5]	5	Correct
6	[6 9 6 4 5 8 6 6]	6	Correct
7	[9 7 9 7 7 9 7]	7	Correct
8	[8 8 5 2 6 3 9 7 4 8 8]	8	Correct
9	[9 7 9 9 6 7 4 9]	9	Correct

The results show that our memory neural network could recall the images it has memorized.

#### 6.2.4. Verification of the Association Ability

**Figure 14:**
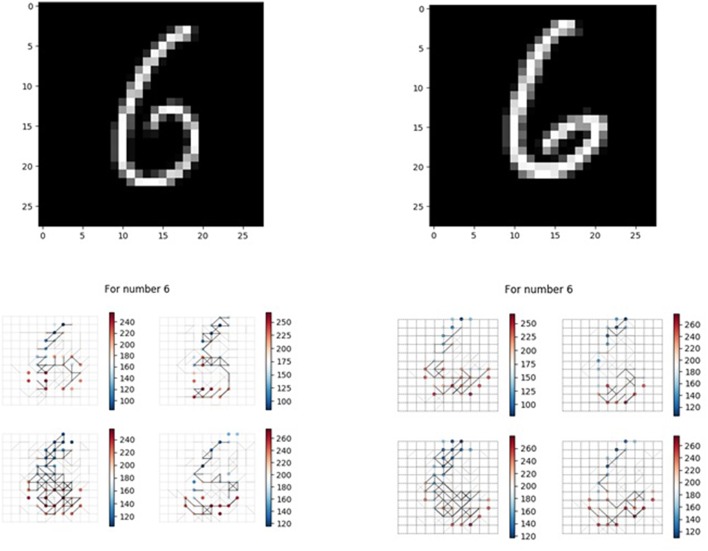
Verification of the association ability.

We also want to test whether, if we feed images that our memory neural network has not seen before but are similar with the images it has memorized, it has the association ability to give a correct result. Figure 14 shows one of the example tests. The 4x different subpanels in each relevant panel correspond to the parts in the memory layer which are the output of each kernel. The memory neural network used is the one generated in the second set of experiments. The left top part is the image used in the process to generate our memory neural network, while the right top image is a new one to test the association ability.

The left bottom part is the recall response of the left top image, while the right bottom part is the response of the memory layer when the new one is fed to the memory neural network. When the left top image is fed to the memory neural network, the firing sequence observed in the output layer is [6 9 6 4 5 8 6 6], and when the right top image is fed to the memory neural network, the firing sequence observed in the output layer is [6 9 4 6]. So when fed with unseen (unmemorized) but similar images, our memory neural network could illustrate some degree of association ability.

## 7. Conclusion

In this paper, we presented our effort at constructing an associative memory neural network through SNNs. We broke the neural network building process into two phases: the Structure Formation Phase and the Parameter Training Phase. The Structure Formation Phase applies a learning method based on Hebb's rule to provoke neurons in the memory layer growing new synapses to connect to neighbor neurons as a response to the specific input spiking sequences fed to the neural network. The aim of this phase is to train the neural network to memorize the specific input spiking sequences. During the Parameter Training Phase, STDP and reinforcement learning are employed to optimize the weight of synapses, to find a way to allow the neural network to recall the memorized specific input spiking sequences.

Results show that, when the input spiking sequences are fed to the input layer of our memory neural network, under the control of the learning method, new connections would grow in the memory layer, and learning the *Threshold* value could be used to control the sparsity of the generated memory layer. Experiments show that our memory neural network was able to memorize different targets and could recall the images it has memorized. Further experimentation showed that when fed with unseen (unmemorized) but similar images, our memory neural network could also illustrate some degree of association ability.

Future work might include: (1)To teach our memory neural network to memorize more complex targets; (2) to enhance our memory neural network's association ability; (3) to grow our memory neural network into a large-scale memory inference system using our method; and (4) the goal of constructing a memory system with causality reasoning nearly the size of a biological brain.

## Data Availability

The datasets generated for this study are available on request to the corresponding author.

## Author Contributions

YD, JL, and YZ were in charge of data curation. WZ and JZ were in charge of formal analysis. HH, YS, and XY were in charge of methodology. MJ and LD were in charge of software. YL was in charge of validation. ZC was in charge of visualization. XY was in charge of writing. HH, ND, and XY were in charge of funding acquisition.

### Conflict of Interest Statement

The authors declare that the research was conducted in the absence of any commercial or financial relationships that could be construed as a potential conflict of interest.
